# Intestinal Dysbiosis and Lowered Serum Lipopolysaccharide-Binding Protein in Parkinson’s Disease

**DOI:** 10.1371/journal.pone.0142164

**Published:** 2015-11-05

**Authors:** Satoru Hasegawa, Sae Goto, Hirokazu Tsuji, Tatsuya Okuno, Takashi Asahara, Koji Nomoto, Akihide Shibata, Yoshiro Fujisawa, Tomomi Minato, Akira Okamoto, Kinji Ohno, Masaaki Hirayama

**Affiliations:** 1 Department of Pathophysiological Laboratory Sciences, Nagoya University Graduate School of Medicine, Nagoya, Japan; 2 Division of Neurogenetics, Center for Neurological Diseases and Cancer, Nagoya University Graduate School of Medicine, Nagoya, Japan; 3 Yakult Central Institute, Tokyo, Japan; 4 Department of School Health Sciences, Aichi University of Education, Kariya, Japan; Hertie Institute for Clinical Brain Research and German Center for Neurodegenerative Diseases, GERMANY

## Abstract

**Background:**

The intestine is one of the first affected organs in Parkinson’s disease (PD). PD subjects show abnormal staining for *Escherichia coli* and α-synuclein in the colon.

**Methods:**

We recruited 52 PD patients and 36 healthy cohabitants. We measured serum markers and quantified the numbers of 19 fecal bacterial groups/genera/species by quantitative RT-PCR of 16S or 23S rRNA. Although the six most predominant bacterial groups/genera/species covered on average 71.3% of total intestinal bacteria, our analysis was not comprehensive compared to metagenome analysis or 16S rRNA amplicon sequencing.

**Results:**

In PD, the number of *Lactobacillus* was higher, while the sum of analyzed bacteria, *Clostridium coccoides* group, and *Bacteroides fragilis* group were lower than controls. Additionally, the sum of putative hydrogen-producing bacteria was lower in PD. A linear regression model to predict disease durations demonstrated that *C*. *coccoides* group and *Lactobacillus gasseri* subgroup had the largest negative and positive coefficients, respectively. As a linear regression model to predict stool frequencies showed that these bacteria were not associated with constipation, changes in these bacteria were unlikely to represent worsening of constipation in the course of progression of PD. In PD, the serum lipopolysaccharide (LPS)-binding protein levels were lower than controls, while the levels of serum diamine oxidase, a marker for intestinal mucosal integrity, remained unchanged in PD.

**Conclusions:**

The permeability to LPS is likely to be increased without compromising the integrity of intestinal mucosa in PD. The increased intestinal permeability in PD may make the patients susceptible to intestinal dysbiosis. Conversely, intestinal dysbiosis may lead to the increased intestinal permeability. One or both of the two mechanisms may be operational in development and progression of PD.

## Introduction

Parkinson’s disease (PD) is a common neurodegenerative disorder in aged individuals. PD is predicted to affect more than 10 million people worldwide by the year 2030 [1]. Postmortem studies of non-PD subjects disclosed incidental α-synuclein-positive Lewy bodies in the gastrointestinal tract, the olfactory system, and the cardiac sympathetic system, which suggests that α-synuclein pathology in PD may start in these tissues [2]. Similarly, in PD, accumulation of α-synuclein in the enteric nervous system could commence 20 years before the onset of degenerative changes in the central nervous system and the associated motor symptoms in PD [3]. In accordance with these observations, the smell test [4] and cardiac meta-iodobenzylguanidine scintigraphy [5] are useful methods to diagnose early PD. In addition, constipation is the most common premotor symptom in PD, and a study of 12 patients with PD revealed that constipation antedated the development of parkinsonian symptoms by an average of 10 or more years in 10 patients [6].

In PD, intestinal permeability is increased and the hyperpermeability is correlated with increased intestinal staining for *Escherichia coli*; nitrotyrosine, a marker for protein oxidation; and α-synuclein [7]. Oxidative stress produced by macrophages in the luminal wall due to a hyperpermeabilized intestinal wall may account for the accumulation of α-synuclein in the intestinal mucosa. As the intestinal microbiota is likely to have a marked effect on the hyperpermeability-induced oxidative stress, the intestinal microbiota may be causally associated with α-synuclein pathology in the enteric nervous system in PD. In healthy humans, intestinal microbiota produce essential nutrients such as vitamins and organic acids, which are absorbed from the intestinal wall and utilized by the gut epithelium [8]. Organic acids produced by intestinal microbiota could also suppress the growth of pathogens in the intestines. In aging, the taxonomic change of bacterial communities is toward a decrease of beneficial bacteria and an increase of harmful bacteria [8]. When harmful bacteria dominate in the intestine because of constipation or other disease processes, essential nutrients are not produced and the harmful substances are increased. These harmful substances may not have an immediate detrimental effect on the host but may partly contribute to development of PD.

Direct evidence supporting the notion that intestinal microbiota determines a clinical phenotype has been recently reported in obesity [9]. Intestinal bacteria obtained from a pair of obese and non-obese individuals in monozygotic twins were implanted in the gut of wild-type mice, and bacteria from obese individuals conveyed significantly greater increases in body mass and adiposity than those from non-obese individuals. Thus, two possible mechanisms are causally associated with PD: the oxidative stress due to intestinal hyperpermeability and an increase in harmful intestinal bacteria with aging. Intestinal microbiota in PD was recently published [10, 11]. In an effort to control for dietary habits and to seek for the association of intestinal microbiota with serum markers, we analyzed intestinal microbiota in PD and healthy cohabitants. We also analyzed serum inflammatory markers (IL-6, TNF-α, high-sensitivity CRP, and lipopolysaccharide [LPS]-binding protein [LBP]); a serum marker for integrity of intestinal epithelium (diamine oxidase [DAO]); a serum marker for adiposity (leptin); anti-Parkinson’s drugs; and motor and mental performances of each patient.

## Materials and Methods

### Study subjects and evaluation methods

All studies were approved by the ethical review committee of the Nagoya University Graduate School of Medicine (approval #2013–0047). We recruited 52 PD patients [21 men and 31 women, 68.9 ± 6.8 years (mean and SD)] from the outpatient clinic of Nagoya University Hospital, as well as from the Aichi Chapter of the Japan Parkinson’s Disease Association. The 52 PD patients were randomly chosen based on the ease of fecal sampling. As controls, we recruited 36 spouses of PD patients in this study (21 men and 15 women, 68.4 ± 9.7 years) who claimed to have no diseases. Stool samples were available from 45 PD patients and 35 controls. Serum samples were available from 47 PD patients and 30 controls. Each of 52 PD patients and 36 controls gave either or both of stool and serum samples. Written informed consents were given from both the patients and the controls. The severities of PD were evaluated using the Hoehn and Yahr scale, the Unified Parkinson’s Disease Rating Scale (UPDRS) parts I-IV, the Mini Mental Sate Examination (MMSE), the Japanese version of the Montreal Cognitive Assessment (MoCA-J) [12], the Frontal Assessment Battery at bedside (FAB), and the Odor Stick Identification Test for the Japanese (OSIT-J) [13]. We recorded stool frequency in a week as a surrogate marker for constipation, because the established constipation score, Rome III [14], is for evaluating diarrhea in ulcerative colitis or irritable bowel syndrome.

### Biochemical assays

The serum levels of high-sensitivity C-reactive protein (hs-CRP) were measured by latex nephelometry in a private laboratory (SRL Laboratory, Nagoya, Japan). The serum levels of interleukin-6 (IL-6), tumor necrosis factor-α (TNF-α), and leptin were measured using the ELISA kits (HS600B, HSTA00D, and DLP00, respectively) from R&D Systems. The serum levels of LPS-binding protein (LBP) and diamine oxidase (DAO) were measured using the ELISA kits from Hycult Biotech (HK315-01) and Immundiagnostik AG (K8500), respectively. LBP binds to LPS, which is contained in the cell wall of Gram-negative bacteria, is increased in response to acute LPS invasion and decreased in chronic LPS invasion [15]. DAO is a marker for intestinal mucosal integrity, and is decreased when the integrity is compromized [16]. We did not measure serum LPS levels.

### Determination of bacterial counts by rRNA-targeted reverse transcription-quantitative PCR

After enrollment into the study, the participants were asked to submit a fresh fecal sample. The fecal sample was placed directly into a tube (~1.0 g/tube) containing 2 mL of RNAlater (an RNA stabilization solution, Ambion) by the participant or the caregiver. The samples were placed in a refrigerator at 4°C and were anonymously transported at 4°C to the Yakult Central Institute. To quantify the bacteria present in the sample, we extracted total RNA fractions from feces by the previously described method [17, 18], and examined the composition of gut microbiota with the Yakult intestinal Flora-SCAN (YIF-SCAN^®^), which exploited RT-quantitative PCR (qPCR) of bacterial 16S or 23S rRNA [19–21]. When we developed YIF-SCAN, we cultured 19 bacterial groups/genera/species and counted the number of bacteria. We also quantified the copy number of 16S or 23S rRNA by RT-qPCR to make a correlation table between RT-qPCR and bacterial counts [17, 18]. Three serial dilutions of the extracted RNA sample were used for RT-qPCR and the threshold cycle values in the linear range of the assay were applied to the standard curve to estimate the numbers of targeted 19 bacterial groups/genera/species. The 19 bacterial groups/genera/species were comprised of (i) six anaerobic species that predominate in our intestine (*Clostridium coccoides* group, *Clostridium leptum* subgroup, *Bacteroides fragilis* group, *Bifidobacterium*, *Atopobium* cluster, and *Prevotella*); (ii) five potential pathogens (*Clostridium perfringens*, *Enterobacteriaceae*, *Enterococcus*, *Staphylococcus*, and *Pseudomonas*), and (iii) eight *Lactobacilli* (*L*. *gasseri* subgroup, *L*. *brevis*, *L*. *casei* subgroup, *L*. *fermentum*, *L*. *plantarum* subgroup, *L*. *reuteri* subgroup, *L*. *ruminis* subgroup, and *L*. *sakei* subgroup) [17, 18]. For the six most prevalent anaerobic species, we estimated the counts of these bacteria in four healthy subjects using YIF-SCAN and quantified the numbers of total intestinal bacteria by hybridization with a generic probe Eub338. The comparison of the counts of the six bacteria and total bacterial count showed that 71.3 ± 9.4% (mean and SD) of total intestinal bacteria were covered by the six predominant bacterial groups/genera/species in YIF-SCAN [18]. We also confirmed that the counts of these six bacteria estimated by YIF-SCAN and FISH were similar with a correlation coefficient of 0.80 [18]. The counts of the five potential pathogens were ~10,000-times lower than those of the six predominant bacteria [18]. We took advantage of RT-qPCR to estimate the counts of these less abundant potential pathogens. Eight *Lactobacilli* were included in YIF-SCAN, because *Lactobacilli* are generally regarded as beneficial bacteria. In our analysis, the eight *Lactobacillus* subgroups are combined together to make a *Lactobacillus* group, and twelve bacterial groups/genera/species are indicated in S2 Table. The counts of eight *Lactobacillus* subgroups are shown in S3 Table.

### Generation of linear regression models to predict disease durations and stool frequencies using intestinal microbiota

In efforts to estimate the contribution of each bacterium on disease duration and constipation, we made linear regression models using the R programming language. The models enabled us to predict disease durations and stool frequencies with the counts of 19 bacterial groups/genera/species. The coefficient of the linear model represents the contribution of each bacterium. Of the 52 PD patients, we had information on stool frequencies in 39 patients and accurate disease durations in 33 patients. Detailed clinical information including disease durations was not available for patients who participated through the Japan Parkinson’s Disease Association. The bacterial counts were converted to Z-scores for each bacterium to normalize contribution of each bacterium in the modeling. To check the multi-colinearity between each bacterial count, variance inflation factor (VIF) of each bacterium was computed and all of them were lower than 10. We first estimated the root mean squared error (RMSE) of the models to predict total UPDRS scores using the leave-one-out cross validation method. RMSE of the models of total UPDRS scores were 52.8, which was much higher than the standard deviation (SD) of 20.4. We similarly calculated RMSE of the models to predict disease durations, and found that RMSE was 7.6 years, which was comparable to SD of 5.4 years. We thus used disease durations in lieu of total UPDRS scores to make a model. Similarly, RMSE of the models to predict stool frequencies was 1.6/week, which was comparable to SD of 1.2/week. After confirming that reasonable models could be generated by the cross validation, all available data were used to generate linear regression models to estimate the effect of each bacterium on disease durations and stool frequencies.

### Statistical analysis

Statistical analyses were conducted using the JMP Pro 11.2.1 (SAS Institute). Data were expressed as the mean ± SD for normally distributed data and median for data with skewed distribution. The Mann-Whitney *U* test and Student *t*-test were used for data analysis. The Spearman correlation analysis was used to determine the associations. The detection rate was analyzed using the Fisher’s exact test. For multiple comparisons of bacterial counts, the false-discovery rate (FDR, *q*-value) was calculated using the Benjamini and Hochberg method.

## Results

### Demographic profiles of the patients and the controls

Males comprised 40.4% of the PD group and 58.3% of the control group, but this difference was not statistically significant (Table 1). The number of subjects taking lactic acid bacteria-containing beverage once a week or more was not different between the two groups. The body mass index (BMI) and the stool frequency per week were lower in the PD patients than the controls. Comparison of the stool frequency with the clinical scores of PD revealed that the stool frequency was negatively correlated with disease duration (*R* = -0.34) and UPDRS2 (*R* = -0.40), and positively correlated with MMSE (*R* = 0.44), MoCA-J (*R* = 0.42), and FAB (*R* = 0.33) in the PD patients (S1 Table), which is in accordance with the notion that constipation deteriorates with progression of PD.

**Table 1:** Characteristics of study subjects.

	Control^a^	PD^a^	*p*
Gender (n)			n.s.^d^
Male	21	21	
Female	15	31	
Total	36	52	
Age (years)	68.4 ± 9.7	68.9 ± 6.8	n.s.^e^
BMI (kg/m^2^)	22.6 ± 3.7	20.2 ± 2.8	< 0.001^e^
Beverage (%)^b^	58.3	69.2	n.s.^d^
Stool frequency (/week)	7.6 ± 4.6	3.1 ± 1.2	< 0.001^e^
IL-6 (pg/mL)	1.2 ± 0.9	1.1 ± 0.8	n.s.^e^
TNF-α (pg/mL)	1.5 ± 1.1	1.3 ± 0.9	n.s.^e^
hs-CRP (ng/mL)	806 ± 917	606 ± 1388	n.s.^e^
Leptin (pg/mL)	3729 ± 3629	2084 ± 2295	< 0.05^e^
LBP (ng/mL)	10140 ± 5061	7785 ± 2406	< 0.01^e^
DAO (ng/mL)	16.8 ± 7.3	19.5 ± 13.0	n.s.^e^
Disease duration (years)	-	9.5 ± 5.4	
Hoehn and Yahr score	-	2.7 ± 0.9	
L-dopa (mg)	-	350 ± 127	
L-dopa equivalent dose (mg)	-	438 ± 181	
^c^UPDRS1 score	-	2.9 ± 2.3	
^c^UPDRS2 score	-	11.7 ± 6.8	
^c^UPDRS3 score	-	25.6 ± 11.8	
^c^UPDRS4 score	-	3.4 ± 2.4	
MMSE score	-	27.8 ± 4.6	
MoCA-J score	-	25.0 ± 4.0	
FAB score	-	15.6 ± 2.3	

^a^Mean and SD are indicated, if applicable.

^b^Percentage of the subjects who drink lactic acid bacteria-containing beverage once a week or more.

^c^UPDRS scores were obtained during the on-phase at the outpatient clinic.

Statistical difference is examined with the Fisher’s exact test^d^ or the Student’s *t*-test^e^. n.s., not significant.

### Laboratory findings

**Fig 1:**
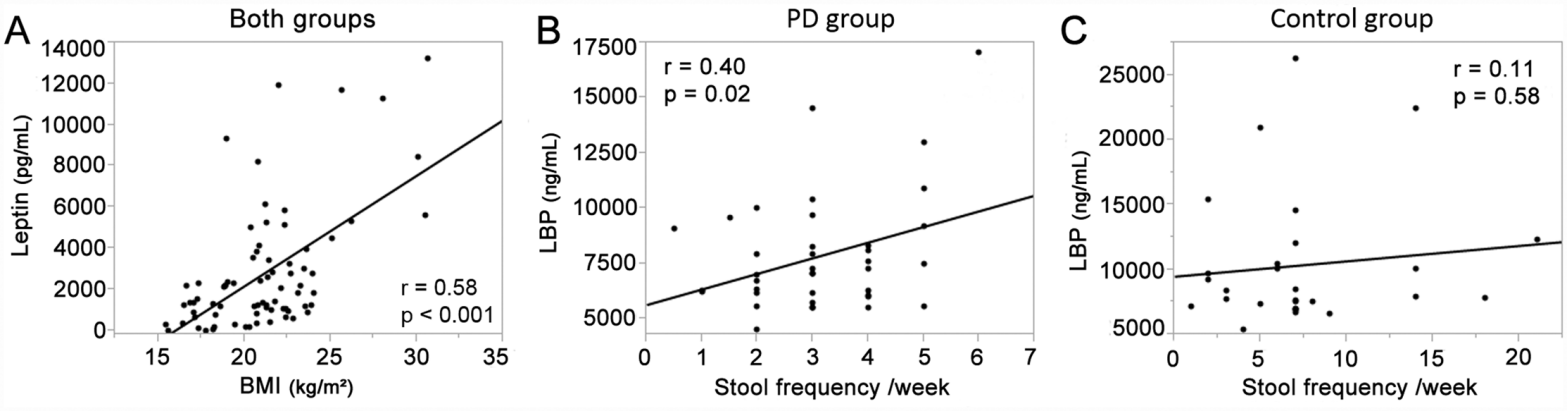
Correlation of serum markers, BMI, and the stool frequency. **(A)** Serum level of leptin was correlated with BMI in the two groups. Serum level of LBP was correlated with stool frequency in the PD group **(B)**, but not in the controls **(C)**.

Serum levels of leptin and LBP were lower in the PD patients compared with the controls (Table 1). On the other hand, serum levels of IL-6, TNF-α, hs-CRP, and DAO were not different between the two groups. The serum level of leptin was significantly correlated with BMI in both groups (Fig 1A). We next analyzed the correlation of stool frequency with laboratory findings in both groups but detected none. In the PD patients, however, the stool frequency was weakly correlated with serum level of LBP, but not with serum levels of leptin, IL-6, TNF-α, hs-CRP, or DAO (Fig 1B and 1C).

### Composition of fecal bacteria

We analyzed fecal bacterial counts of 45 PD patients and 35 cohabitants whose fecal samples were available for our studies. The sum of fecal bacterial counts was lower in PD patients (10.6 ± 0.3 log_10_ cells/g feces) compared to controls (10.7 ± 0.5 log_10_ cells/g feces) with statistical difference (*p* < 0.05 by Mann-Whitney *U* test). The counts of the *Clostridium coccoides* group, *C*. *leptum* subgroup, and *Bacteroides fragilis* group were lower, and the count of *Lactobacillus* was higher in PD patients than controls (Table 2). Six of the eight *Lactobacilli* that we analyzed were higher in PD patients than controls (S3 Table). As the sum of fecal bacterial counts was decreased in PD, we also examined the fractional ratios of each bacterium. Similar to the absolute bacterial counts, the fractional ratios of *C*. *coccoides* group and *B*. *fragilis* group were significantly lower and that of *C*. *leptum* subgroup was slightly lower in the PD patients (data not shown). We also analyzed fecal bacterial counts in available 33 cohabitant pairs to match the sample sizes between PD patients and controls, and obtained similar results (S2 and S4 Tables).

**Table 2:** Comparisons of bacterial counts between control subjects and PD patients.

	Fecal bacterial count (log_10_ cells/g)				Detection rate (%)^a^		
	Control^b^	PD^b^	*p* ^c^	*q* ^d^	Control	PD	*p* ^e^
*C*. *coccoides* group	9.7 ± 0.6	9.3 ± 0.5	2.0E-04*	2.4E-03*	100	100	n.s.
*C*. *leptum* subgroup	10.2 ± 0.6	9.8 ± 1.1	5.8E-03*	2.3E-02*	100	98	n.s.
*B*. *fragilis* group^f^	9.6 ± 0.8	9.3 ± 0.6	9.9E-03*	3.0E-02*	100	100	n.s.
*Bifidobacterium*	9.5 ± 1.2	9.6 ± 1.2	4.7E-01	5.6E-01	100	100	n.s.
*Atopobium* cluster	9.4 ± 0.6	9.5 ± 0.5	8.5E-01	8.5E-01	100	100	n.s.
*Prevotella* ^f^	7.2 ± 2.0	6.7 ± 1.8	2.8E-01	3.7E-01	79	71	n.s.
*C*. *perfringens*	3.6 ± 2.0	3.5 ± 1.5	6.5E-01	7.1E-01	44	58	n.s.
*Lactobacillus*	7.0 ± 1.3	7.8 ± 1.3	3.1E-03*	1.9E-02*	100	100	n.s.
*Enterobacteriaceae* ^f^	7.4 ± 1.2	7.2 ± 1.0	2.5E-01	3.8E-01	97	98	n.s.
*Enterococcus*	6.5 ± 1.4	7.0 ± 1.1	8.2E-02	2.0E-01	94	100	n.s.
*Staphylococcus*	4.7 ± 0.9	4.4 ± 0.9	2.4E-01	3.8E-01	97	96	n.s.
*Pseudomonas* ^f^	3.7 ± 0.8	3.6 ± 0.8	1.8E-01	3.6E-01	32	20	n.s.

^a^Detection rate represents the percentage of fecal samples that contained specific bacterial groups/genera/species above the detection threshold.

^b^Mean and SD are indicated

^c^Statistical difference is examined with Mann-Whitney *U* test.

^d^ *q* value is calculated using the Benjamini and Hochberg method.

^e^Statistical difference is analyzed with Fisher’s exact test.

^f^Gram-negative bacteria. The sum of Gram-negative bacteria in PD (9.5 ± 0.6 log_10_ cells/g) was lower than that in controls (9.9 ± 0.6 log_10_ cells/g) (*p* < 0.001, Mann-Whitney *U* test).

*p or *q* value is less than 0.05.

n.s., not significant.

We first analyzed the effects of anti-PD drugs on intestinal microbiota in PD patients. Correlation coefficients between the daily L-Dopa intake and the counts of 12 intestinal bacteria were from -0.29 to 0.44, and L-Dopa had no effect on the intestinal microbiota. We next compared the counts of 12 intestinal bacteria in patients with or without monoamine oxidases-B, entacapone, pramipexole, ropinirole, zonisamide, anticholinergic agent, or amantadine, and found no statistical difference in any bacteria.

**Fig 2:**
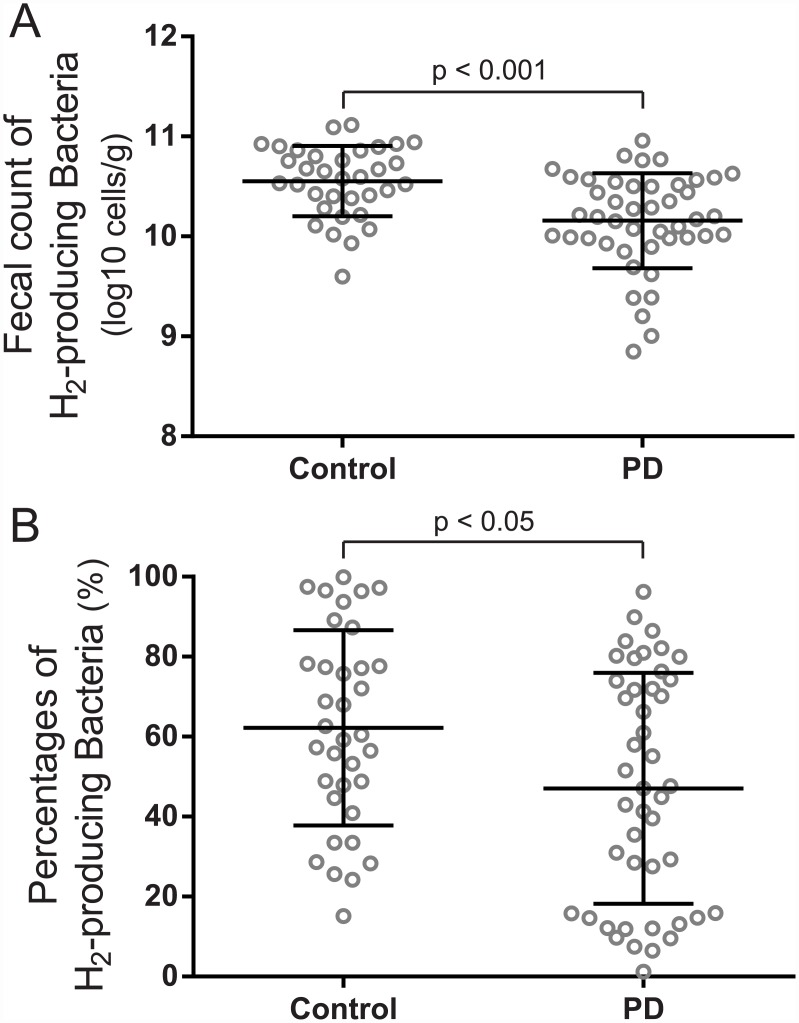
Fecal counts of putative hydrogen-producing bacteria in two groups. The absolute counts **(A)** and the relative ratio **(B)** of hydrogen-producing fecal bacteria were lower in PD patients than that in controls. Mean and SD are indicated. Statistical differences are analyzed by the Mann-Whitney *U* test.

We analyzed the counts of hydrogen-producing bacteria because *per os* administration of hydrogen water is protective against PD in rats [22] and mice [23]. Similarly, hydrogen water (1000 ml/day) improved total UPDRS scores in PD patients in a double-blind randomized controlled study [24]. According to an extensive review of bacterial hydrogenases [25], *B*. *fragilis*, *C*. *perfringens*, and *Pseudomonas* are hydrogen-producing bacteria. Similarly, most strains in family *Enterobacteriaceae* also produce hydrogen. As 12 species in *Clostridium* produce hydrogen, we assumed that *C*. *coccoides* group and *C*. *leptum* subgroup also produce hydrogen. We calculated the sum of these six bacterial groups by assuming that these are hydrogen-producing bacteria, and found that the fecal count and the ratio of putative hydrogen-producing bacteria were significantly lower in PD patients than controls (Fig 2).

### Estimation of the effect of each intestinal bacterium on disease durations and stool frequencies

**Fig 3:**
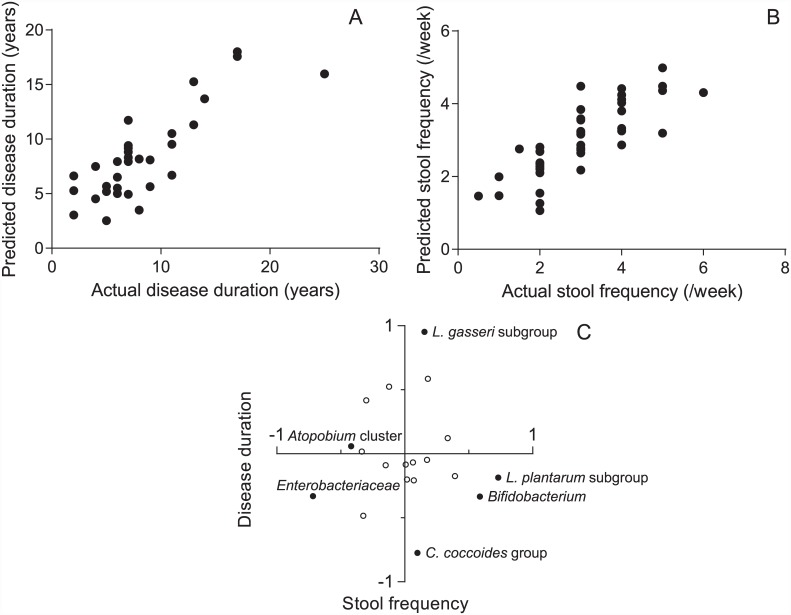
Linear regression models to predict disease durations and stool frequencies. **(A)** Scatter plot of the actual and predicted disease durations. **(B)** Scatter plot of the actual and predicted stool frequencies. **(C)** Scatter plot of the coefficients of each bacterial groups/genera/species to predict disease durations and stool frequencies. Positive and negative coefficients indicate that the bacterial group has a positive and negative effect on disease duration or stool frequency. Higher coefficients indicate higher effects on these parameters. The names of bacterial groups/genera/species that are addressed in discussion are indicated with closed symbols. The coefficients of each bacterial group/genus/species are indicated in S5 Table.

As the PD patients had markedly lower stool frequencies (Table 1), we hoped to distinguish bacterial groups that were associated with disease duration from those associated with constipation. Disease durations are negatively correlated with the loss of dopaminergic neurons in PD [26]. We used disease durations instead of UPDRS scores, because we could not generate a dependable model to predict total UPDRS scores (see Materials and Methods). Although disease duration and constipation correlate each other, some intestinal bacteria may have a marked effect on constipation but not on disease progression. We thus hoped to dissect the effect of each bacterial group on disease progression and constipation. After validating models with the leave-one-out cross validation method (see Materials and Methods), we generated linear regression models to predict disease durations and stool frequencies using intestinal bacterial counts. The Pearson’s correlation coefficient between the predicted and actual disease durations was 0.82 (Fig 3A). Similarly, the Pearson’s correlation coefficient between the predicted and actual stool frequencies was 0.81 (Fig 3B). We next estimated the effects of each bacterium on disease durations and stool frequencies by analyzing the coefficients of the models (Fig 3C, S5 Table). We found that *L*. *gasseri* subgroup had the largest positive coefficient to predict disease durations. In contrast, the coefficient of *L*. *gasseri* subgroup for predicting stool frequencies was close to zero. Thus, the increased counts of *L*. *gasseri* subgroup in PD patients who had longer disease durations were unlikely to be a hallmark of constipation. We similarly found that *C*. *coccoides* group had the largest negative coefficient to predict disease durations. Again, the coefficient of *C*. *coccoides* group for predicting stool frequencies was close to zero, indicating that the decreased counts of *C*. *coccoides* group in PD patients who had longer disease durations is not a hallmark of constipation.

## Discussion

Colonic disorders such as constipation, colonic inflammation, and appearance of α-synuclein in colonic submucosa have been repeatedly reported in PD patients [27–29]. Appearance of intestinal α-synuclein in aged non-PD subjects [27], as well as presymptomatic appearance of intestinal α-synuclein in PD patients [28], imply that the PD pathology may start from the intestine. Although the mechanisms underlying the increased intestinal permeability in PD [7] remain elusive, the increased permeability should make the intestinal neuronal cells sensitive to intestinal microbiota. Three peptidoglycan recognition proteins encoded by the *PGLYRP* genes are essential to maintain healthy gut microbiota by regulating the immune response to both commensal and harmful bacteria. Causal association between intestinal microbiota and PD is also inferred from an observation that single nucleotide polymorphisms in the *PGLYRP* genes are associated with the risk of PD [30]. Additional supporting evidence is that oral administration of LPS is able to induce intestinal PD pathology in rodents [31, 32]. A recent report on the transmission of the obese phenotype from human to mouse using the intestinal microbiota [9] also supports the notion that intestinal bacteria potentially determine a clinical phenotype. Another intestinal abnormality in PD is small intestinal bacterial overgrowth (SIBO), where the bacterial density of small intestine is above 10^5^ colony-forming units/ml and the colonic bacterial species are present in the small intestine [33]. Investigators in Italy reported that the prevalence of SIBO was higher in PD patients, and PD patients with SIBO had longer off-time and more episodes of delayed-on and no-on than those without SIBO [34–36]. Interestingly, the eradication of SIBO with antibiotics resulted in improvement in motor fluctuations without affecting the pharmacokinetics of levodopa [34–36]. Although not directly relevant to intestinal microbiota, it is interesting to note *Helicobacter pylori* infection is associated with worsening of PD [37, 38].

We analyzed 19 bacterial groups/genera/species of intestinal microbiota in PD and their correlations with clinical findings and serum markers (LBP, DAO, IL-6, TNF-α, hs-CRP, and leptin). Leptin is produced by white adipose tissue and the serum level of leptin is positively correlated with the amount of fat in the body [39]. As predicted, BMI and the serum level of leptin were positively correlated in both PD patients and controls, and PD patients had lower BMI’s and lower serum leptin levels than controls (Table 1). Because we recruited cohabitants of PD patients to match the lifestyles between the two groups, the difference in intestinal microbiota in PD patients and controls might represent the difference in BMI’s. Similarly, we could not match the genders and the difference in intestinal microbiota might be due to the difference in the genders. We, however, hoped to control the effects of diet, because diet has a significant effect on intestinal microbiota [40].

We found that the sum of fecal bacteria was lower in PD patients. Our PD patients frequently had constipation (Table 1), which was described even in the first patients reported by Dr. Parkinson [41]. A decreased number of fecal bacteria was previously reported in patients with the constipation-type irritable bowel syndrome [42]. Although the underlying mechanisms are not known, the decreased number of fecal bacteria might simply represent a high frequency of constipation in PD. We found that the absolute counts of *C*. *coccoides* group and *B*. *fragilis* group were lower in PD and those of *Lactobacillus* were higher in PD compared with controls (Table 2). Linear regression models revealed that the increased count of *L*. *gasseri* subgroup was associated with disease duration (Fig 3C). Although constipation gets worse in the course of progression of PD, the increased count of *L*. *gasseri* subgroup was irrelevant to constipation. Similarly, the decreased count of *C*. *coccoides* group was associated with disease duration (Fig 3C), which again was not associated with constipation. *C*. *coccoides* group is a member of obligate anaerobe, and the sum of analyzed obligate anaerobe was indeed decreased in PD (Table 2). In three previous studies, the numbers of obligate anaerobe were decreased in patients with irritable bowel syndrome [42], colorectal cancer [20], and type 2 diabetes [21]. Thus, the decreased counts of obligate anaerobe are unlikely to be unique to PD, but are nonspecifically observed in a variety of diseases.

The linear regression model revealed that *Enterobacteriaceae* and *Atopobium* cluster are negatively, and *L*. *plantarum* subgroup and *Bifidobacterium* are positively, associated with stool frequencies (Fig 3, S5 Table). Although constipation is a frequent symptom in PD, the numbers of these bacteria were similar between PD and controls (Table 2), indicating that these bacteria are associated with constipation but not with PD.

Scheperjans and colleagues recently reported that the fecal count of *Prevotella* was reduced 4.5-fold in PD [10]. Keshavarzian and colleagues similarly demonstrated that *Prevotella* was reduced 2-fold without statistical significance in intestinal mucosa in PD, but was not changed in stools in PD [11]. The count of *Prevotella* was also reduced 3.2-fold in our PD patients (Table 2). Although there was no statistical difference between controls and PD patients, *Prevotella* was the most reduced bacteria in our PD patients. Scheperjans also hypothesized that low *Prevotella* counts might lead to decreased mucin synthesis and increased gut permeability in PD [10]. The decreased LBP without decreasing DAO in our PD patients indeed indicates increased gut permeability and supports their hypothesis. In contrast to *Prevotella*, the count of *Lactobacillus* was increased 22-fold in the previous report [10], and 6.3-fold in our patients (Table 2). The increased counts of *Lactobacillus* were statistically significant in both studies. As the increased *Lactobacillus* was also observed in diabetes mellitus type 2 [21] and the constipation-type irritable bowel syndrome [42], it may not have disease specificity.

We and others have previously reported that hydrogen water prevents development of PD in the 6-OH-DA-induced rat model of PD [22, 43], the MPTP-induced mouse model of PD [23], and PD patients [24]. Most studies on hydrogen in rodents and human including PD have been conducted with *per os* administration of hydrogen water, inhalation of hydrogen gas, or injection of hydrogen saline. The effect of hydrogen-producing intestinal bacteria has been demonstrated only in Concanavarin A (ConA)-induced hepatitis [44], but not in PD. Suppression of intestinal microbiota by antibiotics worsened ConA-induced hepatitis. Reconstitution of intestinal microbiota with hydrogen-producing *E*. *coli*, but not with hydrogen-deficient mutant *E*. *coli*, ameliorated ConA-induced hepatitis. In our PD patients, the fecal count of putative hydrogen-producing bacteria was decreased (Fig 2). Lactulose is a synthetic disaccharide that can be catalyzed only by intestinal bacteria in human, and a large amount of hydrogen is produced by bacterial catalysis of lactulose [45]. An early-phase elevation of breath hydrogen after taking lactulose is a hallmark to diagnose SIBO in the lactulose breath test, but breath hydrogen becomes much higher after lactulose reaches the large intestine. We previously reported that the total amount of breath hydrogen in PD patients was lower than those in healthy controls [43], which is in accordance with our current observation that hydrogen-producing bacteria was lower in PD patients. The decreased intestinal counts of putative hydrogen-producing bacteria may partly account for development of PD. However, the actual amount of hydrogen produced by each bacterium needs to be experimentally determined to draw a definite conclusion.

LBP is a glycoprotein that is produced in the liver and mostly resides in the blood [46–48]. LBP opsonizes LPS, which is the cell wall constituent of Gram-negative bacteria [49]. When LPS goes into the blood, LBP quickly binds to LPS and facilitates its recognition by macrophages. LBP plays a key role in the innate immune response to Gram-negative bacterial challenge [50]. Although acute LPS invasion increases serum levels of LBP, chronic invasion of LPS rather decreases serum levels of LBP [15]. We found that the serum levels of LBP were lower in PD patients than in controls, which was in accordance with a previous report [7]. In addition, serum levels of LBP were positively correlated with stool frequency only in PD patients (Fig 1B). In PD patients, constipation may exacerbate the invasion of LPS. Thus, the lower levels of serum LBP may be caused by increased invasion of Gram-negative bacteria, although we did not measure the serum LPS levels in PD patients. In accordance with this hypothesis, abnormal staining of intestinal mucosa for *E*. *coli*, as well as increased intestinal permeability, were previously reported in PD patients [7]. Although the serum levels of LBP were decreased in our patients, the serum levels of TNF-α, IL-6, and hs-CRP were not elevated. Increased serum inflammatory markers such as IL-6 are commonly observed in PD, as reviewed by Dzamko et al. [51]. Others, however, report that IL-6 is not elevated in PD patients [52]. The mechanisms underlying discordant serum IL-6 levels in different groups remain to be elucidated. We also observed that the serum level of DAO, a marker for intestinal mucosal integrity [16], was not decreased in PD. Although we did not obtain intestinal mucosal biopsies, preserved DAO suggests that the intestinal walls are not damaged in PD. Taken together, our studies suggest that the intestinal permeability is increased in PD, while the intestinal mucosal integrity is preserved. The increased intestinal permeability in PD may make the patients susceptible to alteration in intestinal microbiota. Conversely, intestinal dysbiosis may lead to the increased intestinal permeability. Further studies are required to elucidate the causal associations between intestinal dysbiosis, increased intestinal permeability, and LPS invasion.

## Supporting Information

S1 Table Correlation coefficients between stool frequency and clinical scores. (DOCX)

S2 Table Comparisons of bacterial counts in 33 cohabitant pairs of control subjects and PD patients. (DOCX)

S3 Table Comparisons of *Lactobacillus* counts between control subjects and PD patients. (DOCX)

S4 Table Comparisons of *Lactobacillus* counts in 33 cohabitant pairs of control subjects and PD patients. (DOCX)

S5 Table Coefficients of 19 bacterial groups/genera/species to predict disease durations and stool frequencies with linear regression models. (DOCX)

## Abbreviations

PD: Parkinson’s disease
LPS: lipopolysaccharide
LBP: lipopolysaccharide-binding protein
DAO: diamine oxidase
UPDRS: Unified Parkinson’s Disease Rating Scale
MMSE: Mini Mental Sate Examination
MoCA-J: Japanese version of the Montreal Cognitive Assessment
FAB: Frontal Assessment Battery at bedside
OSIT-J: Odor Stick Identification Test for the Japanese
hs-CRP: high-sensitivity C-reactive protein
IL-6: interleukin-6
TNF-α: tumor necrosis factor-α
YIF-SCAN: Yakult Intestinal Flora-SCAN
qPCR: quantitative PCR
VIF: variance inflation factor
RMSE: root mean squared error
FDR: false-discovery rate
BMI: body mass index
SIBO: small intestinal bacterial overgrowth
ConA: Concanavarin A
