# Supplementary material for: Intestinal Dysbiosis and Lowered Serum Lipopolysaccharide-Binding Protein in Parkinson’s Disease
Source: PLoS One. 2015 Nov 5;10(11):e0142164. doi: 10.1371/journal.pone.0142164 (PMC4634857; doi:10.1371/journal.pone.0142164)
Supplement: S1 Table — (DOCX) [file pone.0142164.s001.docx]

**Supplementary Table 1. Correlation coefficients between stool frequency and clinical scores**

|  | Correlation coefficient (r) with stool frequency |
| --- | --- |
|  |  |
| Disease duration | -0.34 |
| Hoehn and Yahr scale | -0.10 |
| UPDRS1 | -0.07 |
| UPDRS2 | -0.40 |
| UPDRS3 | -0.13 |
| UPDRS4 | -0.10 |
| MMSE | 0.44 |
| MoCA-J | 0.42 |
| FAB | 0.33 |
| OSIT-J (Smell) | -0.27 |
