# Supplementary material for: Intestinal Dysbiosis and Lowered Serum Lipopolysaccharide-Binding Protein in Parkinson’s Disease
Source: PLoS One. 2015 Nov 5;10(11):e0142164. doi: 10.1371/journal.pone.0142164 (PMC4634857; doi:10.1371/journal.pone.0142164)
Supplement: S2 Table — (DOCX) [file pone.0142164.s002.docx]

**Supplementary Table 2. Comparisons of bacterial counts in 33 cohabitant pairs of control subjects and PD patients**

|  | | Fecal bacterial count (log_10_ cells/g) | | | | | Detection rate (%)^a^ | | |
| --- | --- | --- | --- | --- | --- | --- | --- | --- | --- |
|  | Control^b^ | | PD^b^ | *p*^c^ | *q*^d^ | | Control | PD | *p*^e^ |
| *C. coccoides* group | 9.6 ± 0.6 | | 9.2 ± 0.5 | 1.3E-04^*^ | | 1.6E-03^*^ | 100 | 100 | n.s. |
| *C. leptum* subgroup | 10.2 ± 0.6 | | 9.7 ± 1.0 | 1.1E-02^*^ | | 3.3E-02^*^ | 100 | 100 | n.s. |
| *B. fragilis* group^f^ | 9.6 ± 0.8 | | 9.2 ± 0.6 | 7.4E-03^*^ | | 3.2E-02^*^ | 100 | 100 | n.s. |
| *Bifidobacterium* | 9.5 ± 1.2 | | 9.7 ± 1.1 | 3.0E-01 | | 4.7E-01 | 100 | 100 | n.s. |
| *Atopobium* cluster | 9.4 ± 0.7 | | 9.5 ± 0.5 | 4.1E-01 | | 4.9E-01 | 100 | 100 | n.s. |
| *Prevotella*^f^ | 7.2 ± 2.0 | | 9.4 ± 1.4 | 1.3E-01 | | 2.6E-01 | 79 | 67 | n.s. |
| *C. perfringens* | 3.6 ± 2.0 | | 3.5 ± 1.5 | 7.6E-01 | | 8.3E-01 | 45 | 52 | n.s. |
| *Lactobacillus* | 7.0 ± 1.3 | | 7.8 ± 1.4 | 8.0E-03^*^ | | 3.2E-02^*^ | 100 | 100 | n.s. |
| *Enterobacteriaceae*^f^ | 7.4 ± 1.2 | | 7.3 ± 1.0 | 3.4E-01 | | 4.7E-01 | 97 | 100 | n.s. |
| *Enterococcus* | 6.5 ± 1.4 | | 7.1 ± 1.1 | 9.9E-02 | | 2.4E-01 | 93 | 100 | n.s. |
| *Staphylococcus* | 4.7 ± 0.9 | | 4.6 ± 1.0 | 9.4E-01 | | 9.4E-01 | 100 | 94 | n.s. |
| *Pseudomonas*^e^ | 3.8 ± 0.8 | | 3.7 ± 0.8 | 3.5E-01 | | 4.7E-01 | 30 | 24 | n.s. |

^a^Detection rate represents the ratio of fecal samples that contained specific bacterial groups/genera/species above the detection threshold.

^b^Mean and SD are indicated

^c^Statistical difference is examined with Mann-Whitney *U* test.

^d^*q* value was calculated using the Benjamini and Hochberg method.

^e^Statistical difference is analyzed with Fisher’s exact test.

^f^Gram-negative bacteria. The sum of Gram-negative bacteria in PD (9.5 ± 0.6 log_10_ cells/g) was lower than that in controls (9.9 ± 0.6 log_10_ cells/g) (*P* < 0.001, Mann-Whitney *U* test).

**p* or *q* value is less than 0.05.

n.s., not significant.
