# Supplementary material for: Intestinal Dysbiosis and Lowered Serum Lipopolysaccharide-Binding Protein in Parkinson’s Disease
Source: PLoS One. 2015 Nov 5;10(11):e0142164. doi: 10.1371/journal.pone.0142164 (PMC4634857; doi:10.1371/journal.pone.0142164)
Supplement: S3 Table — (DOCX) [file pone.0142164.s003.docx]

**Supplementary Table 3. Comparisons of *Lactobacillus* counts between control subjects and PD patients**

|  | Fecal bacterial count (log10 cells/g) | | | | Detection rate (%)^a^ | | |
| --- | --- | --- | --- | --- | --- | --- | --- |
|  | Control^b^ | PD^b^ | *p*^c^ | *q*^d^ | Control | PD | *p*^e^ |
| *L. gasseri* subgroup | 5.8 ± 1.6 | 6.8 ± 1.6 | 7.4E-03^*^ | 1.5E-02^*^ | 88 | 93 | n.s. |
| *L. brevis* | 2.6 ± 0.9 | 3.1 ± 1.2 | 1.4E-02^*^ | 1.9E-02^*^ | 15 | 42 | n.s. |
| *L. casei* subgroup | 4.1 ± 1.4 | 5.2 ± 1.6 | 1.1E-03^*^ | 8.4E-03^*^ | 41 | 76 | n.s. |
| *L. fermentum* | 4.9 ± 1.4 | 6.0 ± 1.7 | 2.1E-03^*^ | 8.4E-03^*^ | 44 | 78 | n.s. |
| *L. plantarum* subgroup | 3.9 ± 1.7 | 4.3 ± 1.8 | 2.7E-01 | 3.1E-01 | 68 | 73 | n.s. |
| *L. reuteri* subgroup | 5.9 ± 1.8 | 6.9 ± 1.8 | 1.1E-02^*^ | 1.8E-02^*^ | 91 | 96 | n.s. |
| *L. ruminis* subgroup | 4.0 ± 2.0 | 5.7 ± 2.6 | 4.6E-03^*^ | 1.2E-02^*^ | 56 | 76 | n.s. |
| *L. sakei* subgroup | 3.8 ± 1.1 | 4.1 ± 1.6 | 7.0E-01 | 7.0E-01 | 56 | 47 | n.s. |

^a^Detection rate represents the ratio of fecal samples that contained specific bacterial groups/genera/species above the detection threshold.

^b^Mean and SD are indicated

^c^Statistical difference is examined with Mann-Whitney *U* test.

^d^*q* value was calculated using the Benjamini and Hochberg method.

^e^Statistical difference is analyzed with Fisher’s exact test.

**p* or *q* value is less than 0.05.

n.s., not significant.
