# Supplementary material for: Intestinal Dysbiosis and Lowered Serum Lipopolysaccharide-Binding Protein in Parkinson’s Disease
Source: PLoS One. 2015 Nov 5;10(11):e0142164. doi: 10.1371/journal.pone.0142164 (PMC4634857; doi:10.1371/journal.pone.0142164)
Supplement: S4 Table — (DOCX) [file pone.0142164.s004.docx]

**Supplementary Table 4. Comparisons of *Lactobacillus* counts in 33 cohabitant pairs of control subjects and PD patients**

|  | Fecal bacterial count (log10 cells/g) | | | | Detection rate (%)^a^ | | |
| --- | --- | --- | --- | --- | --- | --- | --- |
|  | Control^b^ | PD^b^ | *p*^c^ | *q*^d^ | Control | PD | *p*^e^ |
| *L. gasseri* subgroup | 5.8 ± 1.6 | 6.8 ± 1.7 | 1.2E-02^*^ | 2.9E-02^*^ | 91 | 94 | n.s. |
| *L. brevis* | 2.6 ± 0.9 | 2.8 ± 1.1 | 7.4E-02 | 1.5E-01 | 15 | 36 | n.s. |
| *L. casei* subgroup | 4.1 ± 1.4 | 5.5 ± 1.5 | 5.0E-04^*^ | 6.0E-03^*^ | 45 | 82 | < 0.005 |
| *L. fermentum* | 4.9 ± 1.4 | 6.2 ± 1.7 | 5.6E-03^*^ | 2.9E-02^*^ | 45 | 79 | < 0.05 |
| *L. plantarum* subgroup | 3.9 ± 1.7 | 4.3 ± 1.8 | 3.7E-01 | 6.3E-01 | 67 | 73 | n.s. |
| *L. reuteri* subgroup | 5.9 ± 1.8 | 7.0 ± 1.7 | 1.2E-02^*^ | 2.9E-02^*^ | 91 | 97 | n.s. |
| *L. ruminis* subgroup | 4.0 ± 2.0 | 5.9 ± 2.5 | 7.2E-03^*^ | 2.9E-02^*^ | 61 | 79 | n.s. |
| *L. sakei* subgroup | 3.9 ± 1.1 | 3.7 ± 1.3 | 4.3E-01 | 6.5E-01 | 55 | 39 | n.s. |

^a^Detection rate represents the ratio of fecal samples that contained specific bacterial groups/genera/species above the detection threshold.

^b^Mean and SD are indicated

^c^Statistical difference is examined with Mann-Whitney *U* test.

^d^*q* value was calculated using the Benjamini and Hochberg method.

^e^Statistical difference is analyzed with Fisher’s exact test.

**p* or *q* value is less than 0.05.

n.s., not significant.
