# Supplementary material for: Intestinal Dysbiosis and Lowered Serum Lipopolysaccharide-Binding Protein in Parkinson’s Disease
Source: PLoS One. 2015 Nov 5;10(11):e0142164. doi: 10.1371/journal.pone.0142164 (PMC4634857; doi:10.1371/journal.pone.0142164)
Supplement: S5 Table — (DOCX) [file pone.0142164.s005.docx]

**Supplementary Table 5. Coefficients of 19 bacterial groups/genera/species to predict disease durations and stool frequencies with linear regression models**

|  | Coefficients | |
| --- | --- | --- |
| Bacteria | Disease durations | Stool frequencies |
| *L. gasseri* subgroup | 0.952 | 0.154 |
| *B. fragilis* group | 0.586 | 0.181 |
| *L. ruminis* subgroup | 0.524 | -0.123 |
| *Staphylococcus* | 0.417 | -0.301 |
| *Prevotella* | 0.122 | 0.336 |
| *Atopobium* cluster | 0.059 | -0.420 |
| *L. brevis* | 0.018 | -0.336 |
| *L. fermentum* | -0.048 | 0.174 |
| *C. leptum* subgroup | -0.068 | 0.064 |
| *C. perfringens* | -0.084 | 0.008 |
| *L. sakei* subgroup | -0.089 | -0.149 |
| *Pseudomonas* | -0.174 | 0.393 |
| *L. plantarum* subgroup | -0.185 | 0.730 |
| *L. casei* subgroup | -0.201 | 0.017 |
| *Enterococcus* | -0.208 | 0.071 |
| *Enterobacteriaceae* | -0.331 | -0.718 |
| *Bifidobacterium* | -0.334 | 0.588 |
| *L. reuteri* subgroup | -0.484 | -0.325 |
| *C. coccoides* group | -0.775 | 0.099 |

Z-scores of disease durations and stool frequencies are predicted with Z-scores of log of bacterial counts.
